# From plaque to event: coronary plaque burden and morphology in predicting adverse cardiovascular outcomes

**DOI:** 10.3389/fcvm.2026.1761012

**Published:** 2026-02-02

**Authors:** E. Dvinelis, S. Sakalauske, M. Kapacinskaite, G. Cesnaite, R. Stasilo, G. Vrublevska, J. Bacevicius, A. Tamosiunas, S. Glaveckaite

**Affiliations:** 1 Clinic of Cardiac and Vascular Diseases, Institute of Clinical Medicine, Faculty of Medicine, Vilnius University, Vilnius, Lithuania; 2 Centre of Cardiology and Angiology, Vilnius University Hospital Santaros Klinikos, Vilnius, Lithuania; 3 Department of Radiology, Nuclear Medicine and Medical Physics, Institute of Biomedical Sciences, Vilnius University, Vilnius, Lithuania

**Keywords:** cardiovascular risk factors, coronary plaque, major adverse cardiovascular events, plaque burden, plaque morphology

## Abstract

**Aims:**

This study aimed to identify the association of plaque burden and morphological features, such as burden, length, and composition, with major adverse cardiovascular events (MACE) in populations with very high cardiovascular risk using coronary computed tomography angiography (CTA).

**Methods and results:**

A retrospective cohort study was performed in Lithuania among stable symptomatic patients who underwent coronary CTA. All plaques were manually inspected using plaque analysis software. The study included 772 patients. The mean age was 59.6 ± 9.9 years in men and 65.2 ± 8.7 years in women (*p* < 0.001). Women exhibited relatively greater proportions of densely calcified plaque volume, whereas men demonstrated significantly higher proportions of fibrous, fibrofatty, and necrotic core volumes (all *p* < 0.001). Across cardiovascular risk categories, plaque burden, length, total plaque volume, and plaque dense calcium volume increased progressively from the lowest to the highest risk group (all *p* < 0.001). Among plaque characteristics, after adjusting plaque parameters for age, gender, and cardiovascular risk factors in the multivariable regression analysis, all analysed plaque parameters remained significant predictors of all MACE, with plaque burden, fibrofatty and necrotic core volumes being among the strongest (OR 1.048, 95% CI 1.038–1.059, OR 1.072, 95% CI 1.031–1.115, OR 1.056, 95% CI 1.003–1.111, respectively). Additionally, all analysed plaque characteristics, except fibrous volume, remained significant predictors of non-elective MACE. Plaque burden showed the strongest discrimination for all MACE (AUC 0.68; cut-off ≥17.8%) and non-elective MACE (AUC 0.64; cut-off ≥18.3%), while other plaque characteristics demonstrated modest but statistically significant discrimination value.

**Conclusion:**

This study reveals that plaque morphology provides independent prognostic information. Among the studied very high cardiovascular risk population, patients with non-calcified plaque features–especially those with higher fibrofatty and necrotic core volumes–were the strongest independent predictors of MACE, whereas plaque burden provided the highest discriminatory performance for MACE, suggesting it's complementary role in risk assessment.

## 1 Introduction

Despite significant advances in prevention and treatment, coronary artery disease (CAD) remains the leading cause of morbidity and mortality recognised globally (1). CAD is no longer perceived merely as a luminal obstruction; instead, it is recognised as a dynamic and multifactorial process influenced by chronic inflammation, endothelial dysfunction, and the development of vulnerable atherosclerotic plaques (2). It is important to note that acute coronary syndromes frequently arise from lesions that, while non-obstructive, carry a significant risk of coronary events due to specific morphological features (3). This highlights the limitations of a purely stenosis-based diagnostic approach and underscores the importance of coronary computed tomography angiography (CTA) in comprehensive noninvasive plaque evaluation. Lesion-level quantitative analysis has been shown to predict obstructive disease more accurately than per-patient qualitative assessment, highlighting the significance of plaque morphological characteristics (4). In addition to detecting luminal stenosis, coronary CTA provides a comprehensive evaluation of atherosclerotic plaque burden and composition. It evaluates total plaque volume, low-attenuation plaque, as well as the fibrous and calcified components, and the remodeling index (5). These plaque characteristics provide essential insights into a patient's risk profile, enabling more tailored management strategies to help prevent major adverse cardiovascular events (MACE). For instance, total plaque volume and low-attenuation plaque volume have been independently associated with a higher rate of MACE in individuals with stable CAD (6). Adverse coronary plaque features, such as positive remodeling, low attenuation, or napkin-ring sign, are generally associated with an increased risk of MACE in patients with stable chest pain (7, 8). Additionally, cardiovascular risk factors, such as type 2 diabetes mellitus, influence plaque morphology and volume. Individuals with diabetes tend to have plaques with an increased volume of necrotic core and fibrofatty components, which are markers of increased plaque vulnerability (9). While the qualitative evaluation of coronary plaque provides valuable insights, it lacks reproducibility and remains subjective, highlighting the need for a validated and standardised quantitative plaque analysis (10). Fortunately, multiple studies have demonstrated that plaque morphology is not static but evolves in response to therapy. In patients with CAD, statin treatment has been shown to attenuate plaque progression by reducing the necrotic core volume and enhancing the dense calcium content, resulting in a more stable plaque phenotype (11). Biologic therapies, such as alirocumab, further improve plaque stability by reducing the total plaque burden and decreasing the fibrofatty and necrotic core components, thereby enhancing the therapeutic impact of statins (12). Given the complex nature of plaque vulnerability, a thorough evaluation is essential to improve risk stratification and optimise treatment outcomes.

This study was conducted in Lithuania, a country with one of the highest rates of deaths due to circulatory diseases (including cardiovascular diseases)—54.2%, compared to the overall EU percentage of 32.4% (13, 14). Our population is characterised by a high prevalence of cardiovascular risk factors, all of which contribute to an accelerated atherosclerotic burden (15, 16). The novelty of this analysis lies in its comprehensive, quantitative assessment of coronary plaque burden and morphology using coronary CTA in a very-high–cardiovascular-risk Lithuanian population with long-term follow-up. By simultaneously evaluating plaque burden and detailed plaque composition and deriving outcome-specific cut-off values for both all and non-elective MACE, the study demonstrates their complementary prognostic roles beyond traditional risk-factor–based assessment. This approach enhances the clinical interpretability and translational potential of quantitative plaque analysis beyond existing data derived from predominantly low-to-intermediate cardiovascular risk populations in available trials (4, 8, 17).

## 2 Materials and methods

### 2.1 Study population

A retrospective cohort study was conducted at the Cardiology and Angiology Centre of Vilnius University Hospital Santaros Klinikos. The study protocol received approval from Vilnius Regional Biomedical Research Ethics Committee (2023/9-1538-994).

**Figure 1:**
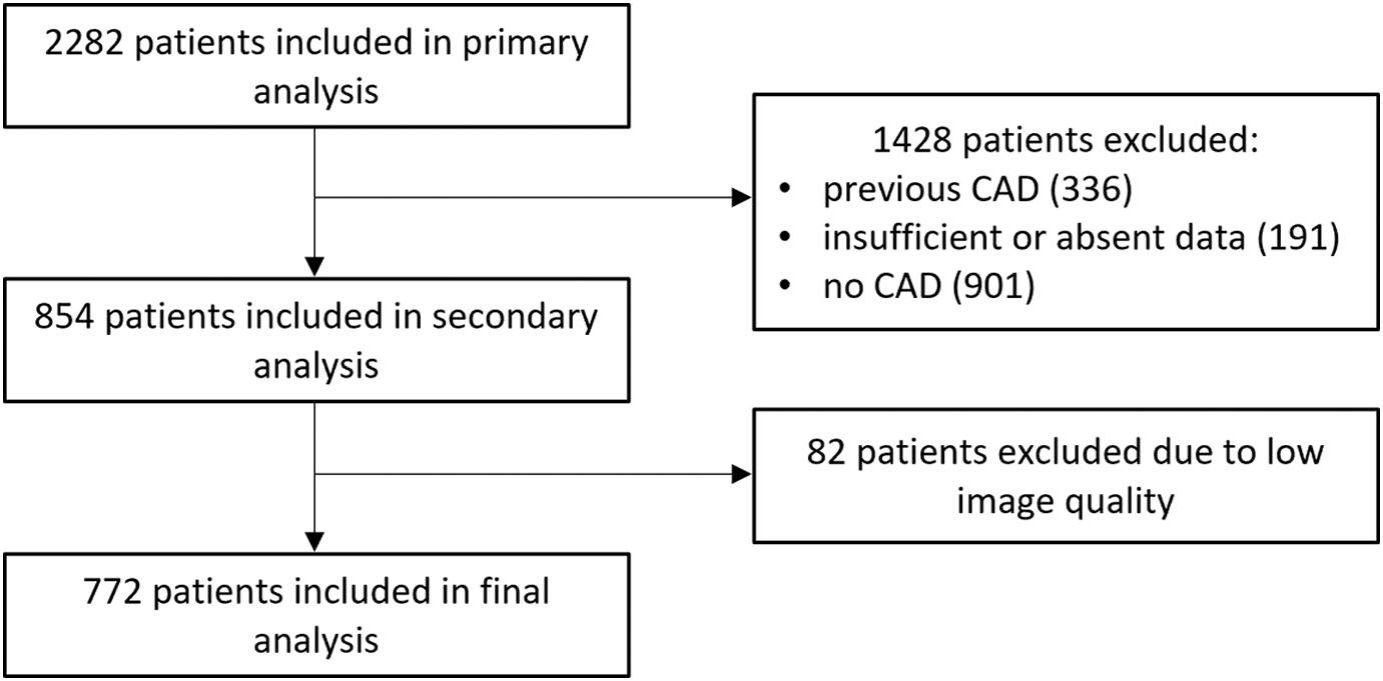
Flow chart of the study. Abbreviations: CAD, coronary artery disease.

An analysis was performed on stable symptomatic patients who underwent coronary CTA due to suspected CAD between January 2018 and December 2019. Participants were excluded if they had pre-existing CAD, which included a history of myocardial infarction (MI), percutaneous coronary intervention, or surgical revascularisation. Additionally, cases with poor coronary CTA image quality (e.g., due to motion artefacts, inadequate contrast enhancement or incomplete visualisation of coronary arteries) were excluded from the analysis. A detailed flow chart is shown in Figure 1.

Data such as demographic and clinical characteristics, cardiovascular risk factors—including both modifiable [smoking, diabetes, hypertension, obesity (BMI) and dyslipidaemia], and non-modifiable (age, gender, and family history)—and symptoms have been collected from electronic health records at the time of referral for coronary CTA. Based on the number of categorical risk factors (smoking, diabetes, hypertension, dyslipidaemia, and family history), a risk score from 0 to 5 was assigned to each patient. The patients were then grouped accordingly and used for stratification to assess changes in plaque morphology across different risk level scores. Outcomes were defined as MACE (acute myocardial infarction, stroke, cardiovascular mortality, and coronary revascularisation). Additionally, we evaluated non-elective MACE (excluding elective coronary revascularisation procedures). Outcomes were assessed at a fixed time point in December 2024 by searching for relevant International Classification of Diseases codes in electronic health records.

### 2.2 Coronary CTA image acquisition and analysis

ECG-triggered coronary CTA images were obtained using a Revolution 256-row detector CT system (GE Healthcare). The scanning parameters included 160 mm of axial coverage, a slice thickness of 0.625 mm, and a gantry rotation time of 280 ms. The tube voltage was adjusted based on the patient's body size.

Oral or intravenous metoprolol was administered to achieve a target heart rate of <65 beats per minute. Data acquisition was triggered using a contrast tracking technique. A non-ionic iodine contrast agent, iopromide (Ultravist 370 mg I/mL), was injected intravenously at a flow rate of 5 mL/sec, with a total volume of 60–80 mL, depending on body mass, followed by a saline flush.

Image post-processing was performed on a dedicated GE workstation and using quantitative plaque analysis software (QAngio CT RE 3.2.14.4, Medis, the Netherlands). Patients with at least one coronary plaque in any vessel, with a diameter greater than 2 mm, were classified as havingcoronary atherosclerosis and were included into analysis. For each case, multiplanar reformations (MPR) were generated along the curved MPR and the centreline of the major epicardial coronary arteries. The vessel centrelines were automatically extracted and manually adjusted to ensure accurate alignment with the arterial lumen. Vessel and lumen boundaries were semi-automatically defined by the software and then manually corrected. Plaque volume was determined by subtracting the lumen volume from the vessel volume within the lesion segment, and plaque length was measured along the vessel centreline; plaque burden was calculated as the proportion of the vessel area occupied by plaque. All plaques were manually inspected using plaque analysis software, with their boundaries carefully outlined by an experienced coronary CTA-board-certified physician, and quantitative analysis of the outlined regions was performed for each lesion. Plaque morphology included: necrotic core [−30–75 Hounsfield units (HU)], fibrofatty (76–130 HU), fibrous (131–350 HU) and dense calcium (>350 HU) components.

### 2.3 Statistical analysis

All data analyses were conducted using SPSS (version 29.0) and Microsoft Excel software. Data were analysed using frequency tables. To test data normality, the Kolmogorov–Smirnov and Shapiro–Wilk tests were used. To compare mean ranks between groups, the Mann–Whitney U test was used. Each plaque characteristic was first examined in univariable logistic regression to assess its association with MACE. Subsequently, a separate multivariable logistic regression analysis was performed, adjusting for age, gender and risk factors, to determine the association between variables and MACE. These relationships were expressed as odds ratios (OR) with corresponding 95% confidence intervals (CI). To visualise differences in multiple plaque parameters across increasing numbers of risk factors, we used radar charts. Each parameter was normalised to a 0–1 scale using min-max scaling to allow comparison despite differences in measurement units. Receiver operating characteristic (ROC) curve analysis was conducted for each plaque characteristic to evaluate its ability to predict the specified outcomes. Discriminatory performance was measured using the area under the curve (AUC) with corresponding 95% confidence intervals. Optimal cut-off values for each variable were identified by maximising the Youden index (sensitivity + specificity—1). Sensitivity and specificity at these thresholds were reported to aid clinical interpretation. A *p*-value of < 0.05 was considered statistically significant.

## 3 Results

### 3.1 Demographic data

Our study included 2,282 patients; 772 met the inclusion criteria and were further analysed. A detailed flow chart is presented in Figure 1. The mean age was 59.6 ± 9.9 years in men and 65.2 ± 8.7 years in women (*p* < 0.001). Most of the study population falls within the 60–80 years age group—61.5% (475 individuals), with 56.6% being male. The baseline characteristics of the study population, categorised by gender, are shown in Table 1. Obstructive coronary lesions (stenosis of ≥50%) were present in 30.8% of the patients. Regarding symptoms, typical angina was reported by 15.9%, and atypical angina by 18.7%; non-anginal symptoms were present in 47.8%, and 17.6% of patients experienced dyspnoea. The most common risk factors were dyslipidaemia and hypertension, with 84% of the patients presenting with two or more risk factors. Significant difference was observed in smoking, which was more prevalent among men (*p* < 0.001); other risk factors showed no significant gender-related differences.

**Table 1:** Baseline characteristics of the study patients by gender.

Characteristics	Men	Women	*p* value
*N*, %	437 (56.6)	335 (43.4)	
Age, ±SD (yrs)	59.6 (9.9)	65.2 (8.7)	<0.001
BMI, ±SD (kg/m^2^)	29 (4.8)	28.8 (4.6)	0.657
Smoker, %	96 (21.5)	25 (7.5)	<0.001
Diabetes, %	384 (13.3)	56 (16.7)	0.181
Family history, %	73 (16.7)	66 (19.7)	0.283
Hypertension, %	384 (87.9)	292 (87.2)	0.768
Dyslipidaemia, %	384 (87.9)	301 (89.9)	0.389
Typical angina	62 (14.2)	61 (18.2)	0.157
Atypical angina	83 (19.0)	61 (18.2)	0.854
Non-anginal pain	229 (52.4)	140 (41.8)	0.004
Dyspnoea	63 (14.4)	73 (21.8)	0.010
	median (IQR)	median (IQR)	
Plaque length (mm)	7.78 (5.09–13.32)	7.00 (4.61–10.95)	<0.001
Plaque burden mean (%)	0.23 (0.16–0.31)	0.25 (0.17–0.32)	0.086
Plaque volume (mm^3^)	21.38 (10.95–48.15)	17.75 (9.61–37.55)	<0.001
Plaque fibrous volume (mm^3^)	8.78 (4.03–17.95)	6.46 (3.09–13.09)	<0.001
Plaque fibrofatty volume (mm^3^)	0.81 (0.25–2.04)	0.39 (0.09–13.09)	<0.001
Plaque necrotic core volume (mm^3^)	0.22 (0.03–0.84)	0.09 (0.00–0.40)	<0.001
Plaque dense calcium volume (mm^3^)	10.41 (3.88–27.78)	9.63 (4.47–21.98)	0.338
Plaque fibrous volume (%)	39.15 (27.99–52.43)	34.32 (23.11–50.68)	<0.001
Plaque fibrofatty volume (%)	2.94 (1.16–6.69)	1.77 (0.63–4.30)	<0.001
Plaque necrotic core volume (%)	0.80 (0.09–2.61)	0.41 (0.00–1.74)	<0.001
Plaque dense calcium volume (%)	55.00 (36.02–68.39)	60.74 (43.38–74.45)	<0.001

BMI, body mass index; IQR, interquartile range; *N*, number of patients; SD, standard deviation.

### 3.2 Plaque burden and characteristics in relation to cardiovascular risk and gender

A total of 2,100 plaques were analysed. When stratified by gender, men demonstrated higher total plaque volumes compared with women [21.38 mm^3^ (10.95–48.15) vs. 17.75 mm^3^ (9.61–37.55), *p* < 0.001]. The same tendency was observed across all plaque parameters, except for plaque burden (*p* = 0.086) and dense calcium volume (*p* = 0.338). In contrast, women exhibited relatively greater proportions of densely calcified plaque volume [60.74% (43.38–74.45) vs. 55.00% (36.02–68.39), *p* < 0.001], whereas men demonstrated significantly higher proportions of fibrous, fibrofatty, and necrotic core volumes (all *p* < 0.001). The detailed parameters of plaque characteristics across genders are shown in Table 1.

Additionally, plaque parameters were analysed in relation to cardiovascular risk score categories. Detailed plaque characteristics for each risk factor score group are shown in Table 2. Across cardiovascular risk categories, plaque burden [0.12 (0.09–0.19) vs. 0.24 (0.16–0.31), *p* < 0.001], length [5.96 (4.95–6.76) vs. 10.48 (5.75–15.36), *p* < 0.001], total plaque volume [9.50 (7.03–14.19) vs. 29.53 (14.47–57.76), *p* < 0.001] and plaque dense calcium volume (6.39 (3.55–9,69) vs. 16.27 (5.94–42.41) increased progressively from the lowest to the highest risk group. Radar plot analysis (Supplementary Figure 1) demonstrated that patients with a higher amount of cardiovascular risk factors exhibited a greater burden across multiple plaque characteristics.

**Table 2:** Plaque characteristics in patients across different risk scores.

Characteristics	0	1	2	3	4	5	*p* value
N, %	13 (1.7)	104 (13.5)	409 (53.0)	181 (23.4)	57 (7.4)	8 (1.0)	
Plaque length (mm)	5.96 (4.95–6.76)	6.30 (4.56–9.32)	7.18 (4.93–11.32)	8.74 (5.22–16.27)	7.73 (5.59–14.34)	10.48 (5.75–15.36)	<0.001
Plaque burden (%)	0.12 (0.09–0.19)	0.19 (0.13–0.27)	0.22 (0.16–0.29)	0.25 (0.18–0.36)	0.27 (0.19–0.35)	0.24 (0.16–0.31)	<0.001
Plaque volume (mm^3^)	9.50 (7.03–14.19)	13.39 (7.87–29.22)	19.86 (10.36–39.14)	25.21 (11.29–67.65)	23.53 (12.73–49.54)	29.53 (14.47–57.76)	<0.001
Plaque fibrous volume (mm^3^)	3.63 (2.49–4.78)	5.47 (3.06–11.98)	7.48 (3.81–14.43)	9.50 (4.52–23.62)	9.43 (4.22–18.43)	10.06 (5.95–22.16)	<0.001
Plaque fibrofatty volume (mm^3^)	0.14 (0.06–0.31)	0.47 (0.12–1.11)	0.56 (0.16–1.46)	0.94 (0.25–2.47)	0.65 (0.20–2.09)	0.50 (0.19–1.12)	<0.001
Plaque necrotic core volume (mm^3^)	0.06 (0–0.13)	0.09 (0–0.41)	0.16 (0–0.59)	0.31 (0.03–2.47)	0.22 (0–0.87)	0.11 (0–0.55)	<0.001
Plaque dense calcium volume (mm^3^)	6.39 (3.55–9.69)	7.06 (3.09–14.68)	10.05 (4.01–23.71)	11.25 (4.23–37.22)	13.47 (5.49–31.83)	16.27 (5.94–42.41)	<0.001

*N*, number of patients; values are shown as medians and interquartile ranges (IQR).

### 3.3 Association between plaque morphology and MACE

**Figure 2:**
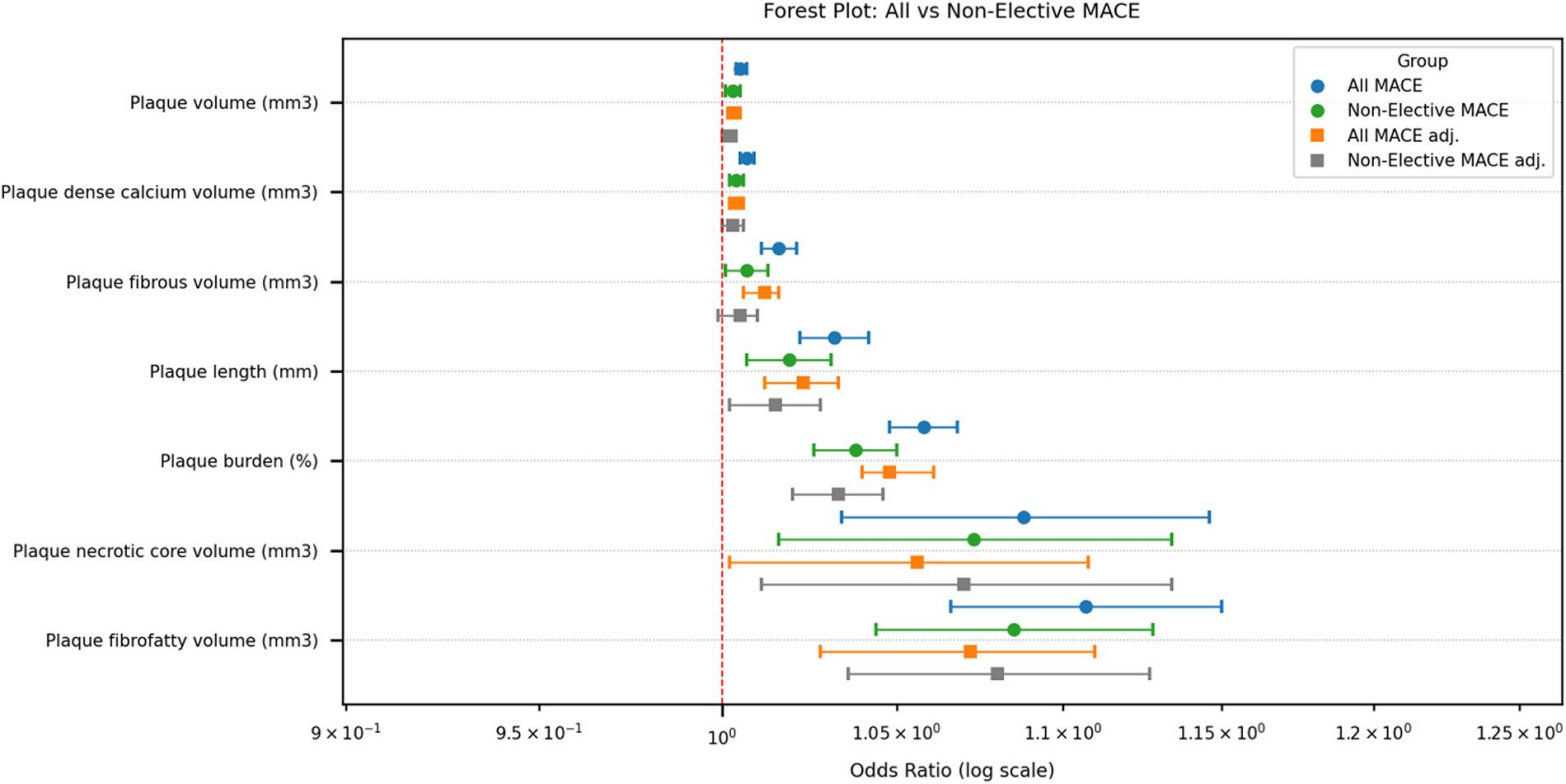
Forest plot of odds ratios for plaque characteristics associated with all and non-elective MACE. Abbreviations: MACE, major adverse cardiovascular events; adj, adjusting for age, gender and risk factors, to determine the association between variables and MACE.

During a median follow-up of 70 months (5.8 years) 111 patients (14.4%) experienced MACE (2.5% of patients with MACE annually): 53 (48%) elective coronary revascularisations, 40 (36%) non-fatal MI or unstable angina, 11 (10%) stroke, and 7 (6%) cardiovascular deaths. Of all MACE, 69 (62%) were in men and 42 (38%) in women. After excluding elective procedures, 58 non-elective MACE events remained, comprising 34 (59%) men and 24 (41%) women. Significant differences were observed in all analysed plaque characteristics between groups with all and non-elective MACE and without MACE; the results are presented in Table 3. Among plaque characteristics, using univariable logistic regression analysis (Table 4), plaque length, burden, volume, fibrous, fibrofatty, necrotic core, dense calcium, and non-calcified plaque volumes were all significantly associated with all and non-elective MACE. Strongest predictors of all MACE were plaque length, plaque burden, plaque fibrofatty, and necrotic core volume (OR 1.032, 95% CI 1.022–1.042, OR 1.058, 95% CI 1.048–1.068, OR 1.107, 95% CI 1.066–1.150, and OR 1.088, 95% CI 1.034–1.146, respectively). Considering non-elective MACE, all analysed plaque parameters were significant predictors for MACE, with plaque burden, fibrofatty and necrotic core volumes being among the strongest (OR 1.038, 95% CI 1.026–1.050, OR 1.085, 95% CI 1.044–1.128, OR 1.073, 95% CI 1.016–1.134, respectively). The results are presented in Table 4. After adjusting plaque parameters for age, gender, and cardiovascular risk factors in the multivariable logistic regression analysis, all analysed plaque parameters remained significant predictors of all MACE (Table 5). Additionally, all analysed plaque characteristics except for fibrous volume remained significant predictors of non-elective MACE; detailed data are presented in Table 5 and a forest plot (Figure 2). Plaque content was also analysed as percentages; an important difference between groups (with and without adverse effects) was identified for necrotic core volume in all MACE (2.2% vs. 2.5%, *p* = 0.024) and non-elective MACE (2.3% vs. 2.5%, *p* = 0.039). In contrast, other parameters did not differ; these results are provided in Supplementary Table 1.

**Table 3:** Plaque characteristics in patients with all and non-elective MACE versus patients without MACE.

Characteristics	All MACE		*p* value	Non-Elective MACE		*p* value
*N*, %	111 (14.4)			58 (7.5)		
	No	Yes		No	Yes	
Plaque length (mm)	7.01 (4.80–11.05)	9.63 (5.65–17.87)	<0.001	7.34 (4.93–11.83)	8.98 (5.06–17.76)	0.004
Plaque burden mean (%)	0.21 (0.15–0.29)	0.29 (0.21–0.38)	<0.001	0.22 (0.15–0.31)	0.27 (0.21–0.36)	<0.001
Plaque volume (mm^3^)	18.21 (9.72–38.36)	30.64 (14.20–72.95)	<0.001	19.70 (10.22–41.62)	27.37 (13.03–76.44)	<0.001
Plaque fibrous volume (mm^3^)	7.00 (3.57–14.19)	11.91 (5.38–24.14)	<0.001	7.54 (3.77–15.66)	9.56 (4.31–21.37)	0.003
Plaque fibrofatty volume (mm^3^)	0.55 (0.16–1.41)	1.08 (0.31–2.41)	<0.001	0.58 (0.16–1.53)	0.97 (0.26–2.34)	<0.001
Plaque necrotic core volume (mm^3^)	0.15 (0–0.56)	0.34 (0.03–1.09)	<0.001	0.16 (0–0.60)	0.28 (0.03–1.21)	<0.001
Plaque dense calcium volume (mm^3^)	8.94 (3.79–21.91)	16.06 (5.53–42.15)	<0.001	9.63 (3.9–24.06)	14.46 (5.31–43.97)	<0.001

MACE, major adverse cardiovascular events; *N*, number of patients; values are shown as medians and interquartile ranges (IQR).

**Table 4:** Plaque characteristics for prediction of MACE (univariable logistic regression analysis).

Predictor	All MACE OR (95% CI)	*p*-value	Non-Elective MACE OR (95% CI)	*p*-value
Plaque length	1.032 (1.022–1.042)	<0.001	1.019 (1.007–1.031)	0.002
Plaque burden	1.058 (1.048–1.068)	<0.001	1.038 (1.026–1.050)	<0.001
Plaque volume	1.005 (1.004–1.007)	<0.001	1.003 (1.001–1.005)	0.001
Plaque fibrous volume	1.016 (1.011–1.021)	<0.001	1.007 (1.001–1.013)	0.014
Plaque fibrofatty volume	1.107 (1.066–1.150)	<0.001	1.085 (1.044–1.128)	<0.001
Plaque necrotic core volume	1.088 (1.034–1.146)	0.001	1.073 (1.016–1.134)	0.012
Plaque dense calcium volume	1.007 (1.005–1.009)	<0.001	1.004 (1.002–1.006)	0.001
Non-calcified plaque volume	1.014 (1.010–1.019)	<0.001	1.007 (1.002–1.012)	<0.001

CI, confidence interval; MACE, major cardiovascular adverse events; OR, odds ratio, non-calcified plaque volume (total plaque volume minus calcified plaque volume).

**Table 5:** Plaque characteristics for prediction of MACE (multivariable logistic analysis after adjustments for age, gender and cardiovascular risk factors).

Predictor	All MACE OR (95% CI)	*p*-value	Non-elective MACE OR (95% CI)	*p*-value
Plaque length	1.023 (1.013–1.034)	<0.001	1.015 (1.002–1.028)	0.024
Plaque burden	1.048 (1.038–1.059)	<0.001	1.033 (1.020–1.046)	<0.001
Plaque volume	1.003 (1.002–1.005)	<0.001	1.002 (1.000–1.004)	0.034
Plaque fibrous volume	1.012 (1.006–1.017)	<0.001	1.005 (0.999–1.011)	0.105
Plaque fibrofatty volume	1.072 (1.031–1.115)	<0.001	1.080 (1.036–1.127)	<0.001
Plaque necrotic core volume	1.056 (1.003–1.111)	0.037	1.070 (1.011–1.134)	0.02
Plaque dense calcium volume	1.004 (1.002–1.006)	<0.001	1.003 (1.000–1.006)	0.03
Non-calcified plaque volume	1.010 (1.005–1.014)	<0.001	1.005 (1.000–1.010)	0.047

CI, confidence interval; MACE, major adverse cardiovascular events; OR, odds ratio, non-calcified plaque volume (total plaque volume minus calcified plaque volume).

For the prediction of all MACE, plaque burden demonstrated the highest discriminatory performance (AUC 0.677), followed by plaque volume and plaque fibrous volume (both AUC 0.625) (Table 6). Plaque burden showed high sensitivity (84.2%) but modest specificity (35.8%) at the optimal cut-off value. In contrast, plaque length, fibrofatty, fibrous, necrotic core, and overall volume provided more balanced profiles of sensitivity and specificity. All plaque characteristics were statistically significant predictors of all MACE (*p* < 0.001). For non-elective MACE, the overall discriminatory performance was lower across all plaque features. Plaque burden again demonstrated the strongest predictive value (AUC 0.635), with high sensitivity (81.8%) and moderate specificity (64.4%). Other plaque characteristics showed modest AUCs (ranging from 0.560 to 0.582) but remained statistically significant. The cut-off values for non-elective MACE were generally higher than those for all MACE, particularly for plaque length, total, necrotic core and dense calcium plaque volumes.

**Table 6:** ROC analysis of coronary plaque characteristics and their cut-offs for prediction of MACE.

A. Outcome: All MACE					
Feature	AUC (95% CI)	*p*-value	Optimal cut-off^a^	Sens. (%)	Spec. (%)
Plaque length (mm)	0.607 (0.576–0.638)	<0.001	≥11.46	42.8	76.6
Plaque burden (%)	0.677 (0.649–0.705)	<0.001	≥17.83	84.2	35.8
Plaque volume (mm^3^)	0.625 (0.596–0.655)	<0.001	≥27.21	55.0	65.0
Plaque fibrous volume (mm^3^)	0.625 (0.595–0.655)	<0.001	≥8.42	63.0	57.7
Plaque fibrofatty volume (mm^3^)	0.610 (0.580–0.640)	<0.001	≥1.19	48.1	70.7
Plaque necrotic core volume (mm^3^)	0.590 (0.560–0.620)	<0.001	≥0.32	51.0	64.9
Plaque dense calcium volume (mm^3^)	0.603 (0.572–0.634)	<0.001	≥5.5	75.9	34.9
B. Outcome: non-elective MACE					
Feature	AUC (95% CI)	*p*-value	Optimal cut-off^a^	Sens. (%)	Spec. (%)
Plaque length (mm)	0.560 (0.517–0.604)	0.007	≥13.97	33.6	80.6
Plaque burden (%)	0.635 (0.597–0.674)	<0.001	≥18.29	81.8	64.4
Plaque volume (mm^3^)	0.578 (0.535–0.620)	<0.001	≥51.10	32.7	80.5
Plaque fibrous volume (mm^3^)	0.561 (0.520–0.603)	0.004	≥8.59	57.9	55.5
Plaque fibrofatty volume (mm^3^)	0.582 (0.541–0.624)	<0.001	≥1.19	47.7	68.3
Plaque necrotic core volume (mm^3^)	0.570 (0.528–0.611)	0.001	≥0.62	36.9	75.2
Plaque dense calcium volume (mm^3^)	0.574 (0.532–0.617)	0.001	≥12.63	58.1	41.9

AUC, area under the curve; MACE, major adverse cardiovascular events; Sens., sensitivity; Spec., specificity.

a Optimal cut-offs determined by maximization of the Youden index.

## 4 Discussion

### 4.1 Determinants of coronary plaque morphology: clinical and demographic factors

Our retrospective cohort study revealed, that among stable symptomatic patients without pre-existing CAD referred to non-invasive coronary imaging by CT and followed up to median 70 months: (a) male gender is associated with bigger plaque volumes and length, as well with more vulnerable plaque characteristics as compared with woman; (b) there is aassociation between plaque burden, vulnerable and more stable plaque characteristics, and the number of cardiovascular risk factors; (c) plaque burden and vulnerability increase the risk of MACE; (d) plaque burden with cut-off value of around 18%was the strongest predictor of all and non-elective MACE among analysed plaque features.

In our study, we observed marked differences in plaque burden and characteristics across gender and cardiovascular risk profiles. The mean age of male participants was significantly lower compared to women [59.6 (9.9) vs. 65.2 (8.7), *p* < 0.001]. This aligns with the concept that male gender is associated with an earlier onset of atherosclerosis, due to higher cumulative exposure to risk factors and hormonal differences (18). Gender was a significant determinant of plaque distribution and type; for instance, men had larger plaques in total volume and a higher overall plaque burden, consistent with prior large-scale studies, including the CONFIRM registry, which demonstrated that male patients have more extensive coronary atherosclerosis than women (17). Notably, our study revealed marked differences in the proportions of plaque components. Men exhibited a higher proportion of fibrofatty and necrotic core plaque volume. These elements are considered more vulnerable to rupture and associated with a higher risk of adverse cardiovascular events. In contrast, women showed a relatively greater proportion of calcified plaque volume, which is generally more stable and less prone to rupture (19). Similarly, a recent study demonstrated that women had a relatively higher proportion of calcified plaque volume than men, despite having a lower overall plaque burden, which may reflect gender-specific differences in plaque morphology and partly explain the lower incidence of adverse events in women at similar levels of atherosclerotic burden (20).

Differences in plaque composition were also evident across the risk categories. Our observation aligns with prior imaging-based studies, which have also noted more prominent adverse plaque features in patients with higher cumulative cardiovascular risk (8)**.** Overall, there was a clear stepwise increase in total plaque burden and in several plaque components with higher risk scores; however, some parameters showed minor deviations, which could be explained by patients receiving intensive risk-factor modification, while those in the higher-risk group are more likely to receive medical therapy (11). Moreover, the highest risk score group (score = 5) consisted of only eight patients, which may limit the statistical reliability of comparisons involving this subgroup; however, the general pattern remained consistent. These findings emphasise that cardiovascular risk scores are closely linked to plaque composition, as higher scores are associated with more vulnerable plaque profiles.

The observed differences in gender-related and risk-factor profiles in plaque composition highlight pathophysiological variations in coronary atherosclerosis, underscoring the need for comprehensive cardiovascular risk assessment, early detection strategies, and targeted prevention.

### 4.2 Plaque morphology as a predictor of major adverse cardiovascular events

In this study, we investigated the relationship between coronary plaque morphology and MACE. Our results indicate that plaque phenotype, rather than stenosis severity alone, influences future events. The primary aim was to evaluate quantitative plaque measurements, which are more reproducible, and to compare them between groups. A recent meta-analysis indicated that high-risk plaque features are associated with substantially higher MACE risk (21). These findings are supported by a more recent study, which confirms the association between greater total and non-calcified plaque volumes and cardiovascular adverse events (22). After our cohort analysis, we found that plaque fibrofatty and necrotic core volumes were more strongly associated with all and non-elective MACE than other features on univariable and multivariable analysis. Quantitative analysis of plaque composition further demonstrated that MACE-related plaques contained a higher proportion of necrotic core volume, underscoring the plaque vulnerability rather than just plaque burden. Our data suggest that morphological features and plaque composition, especially fibrofatty and necrotic core volumes, provide independent, clinically meaningful prognostic information. This pattern is consistent with prior studies, which was also identified by Lozeratti et al.'s research that emphasises the importance of identifying higher risk plaques (23). Furthermore, the analysis by Stone et al. also demonstrated that MACE during follow-up was equally attributable to both culprit and nonculprit lesions; notably, many of the nonculprit lesions were angiographically mild but exhibited high-risk features as assessed by intravascular imaging (24). These insights emphasise that plaque composition rather than stenosis severity might better predict future events.

### 4.3 Clinical implications: from risk stratification to personalised prevention

In the present analysis, plaque burden cut-offs of approximately 18% yielded high sensitivity for both all MACE (84.2%) and non-elective MACE (81.8%), with AUCs of 0.68 and 0.64, respectively, which are comparable to previously reported CTA-based discriminatory performances for plaque burden, where AUC of 0.71 has been described (25). In addition to total plaque burden, other studies, such as those by Williams MC et al., reported threshold-based associations, where the threshold for low-attenuation plaque burden was 4%; patients with this threshold were nearly five times more likely to suffer a myocardial infarction (19). However, there is a lack of comparable data due to wide variation in imaging acquisition protocols, plaque quantification methods, and outcome definitions across studies, which has limited the generalisability of any single cut-off value, highlighting the importance of reporting study-specific thresholds alongside their discriminatory performance. Deriving outcome-specific cut-off values for coronary CTA plaque metrics is crucial for translating quantitative plaque analysis into clinical practice, as absolute thresholds enable comparisons across different studies.

Our study findings can be considered in three major perspectives: diagnostic refinement, therapeutic management and implications for treatment choices.

Considering the diagnostic approach, this research highlights the diagnostic value of plaque morphology in revealing lesion vulnerability that is not captured by traditional clinical scores or stenosis-based assessment alone. Clinical trials support the clinical impact of coronary CTA-guided care: in the SCOT-HEART trial, adding coronary CTA to standard care lowered the 5-year coronary death and nonfatal MI, mainly through more effective preventive therapies in patients with CAD, particularly those with high-risk plaques (26). Other clinical trials highlight the importance of aggressive lipid-lowering therapies. For example, statins have demonstrated a reduction in lipid-rich components and promote a more fibrotic and calcified plaque phenotype, while PCSK9 inhibitors have shown regression of atheroma burden (11, 27). These findings suggest that recognising plaque morphology may influence therapeutic management, including the selection of appropriate invasive or non-invasive approaches and the optimisation of preventive treatment. However, despite its diagnostic capabilities, this comprehensive assessment is relatively time-consuming and may limit its routine clinical use.

## 5 Limitations and strengths

This research was a retrospective cohort study, which might have overlooked some relevant clinical variables. Furthermore, we did not include data on patients’ medication use. While statins are known to influence plaque morphology, evaluating their impact would have been challenging due to the poor compliance in usage in our population and incomplete documentation in medical records. Additionally, some residual confounders may not be entirely excluded despite our efforts to adjust for significant risk factors, which could support our results in the prospective analysis.

Although single centre study, it included a relatively large patient cohort with balanced representation between genders, which enhances the generalizability of our findings. Additionally, we performed a comprehensive three-dimensional assessment of each plaque manually, enabling a detailed and objective evaluation of plaque morphology and minimising potential errors in automated evaluation. One of the key strengths of the present study is the derivation of outcome-specific cut-off values for multiple coronary CTA–derived plaque metrics. These thresholds enhance clinical interpretability, providing a structured framework for future validation and incorporation into multivariable risk prediction models.The technological advances should be taken into account as the newest computed tomography technologies enable more precise evaluation of plaque morphological features.

## 6 Conclusion

Our analysis reveals that plaque morphology provides measurable, independent prognostic information beyond conventional demographic and risk-factor-based models. Among the studied very-high-cardiovascular-risk Lithuanian population, non-calcified plaque features (especially higher fibrofatty and necrotic core volumes) were the strongest independent predictors of all and non-elective MACE. Additionally, risk factor scores were associated with increased volumes across all plaque components, as well as greater total plaque burden. Plaque burden demonstrated the most consistent discriminatory performance for MACE. Although individual plaque features showed only modest predictive accuracy, the identification of clinically relevant cut-off values underscores their potential utility in risk stratification. The study's findings inform future research on the relationship between plaque morphology and clinical events and support the development of standardized assessment. Integrating plaque metrics with clinical variables may further improve prediction of adverse cardiovascular outcomes.

## Data Availability

The raw data supporting the conclusions of this article will be made available by the authors, without undue reservation.
