# Supplementary material for: From plaque to event: coronary plaque burden and morphology in predicting adverse cardiovascular outcomes
Source: Front Cardiovasc Med. 2026 Feb 2;13:1761012. doi: 10.3389/fcvm.2026.1761012 (PMC12907141; doi:10.3389/fcvm.2026.1761012)
Supplement: Supplementary file 1 [file Table1.docx]

**Supplementary Table 1. Plaque characteristics expressed in percentages in patients with versus without MACE**

|  | **All MACE** | | **p value** | **Non-Elective MACE** | | **p value** |
| --- | --- | --- | --- | --- | --- | --- |
| **N, %** | 111 (14.4) | |  | 58 (7.5) | |  |
|  | **No** | **Yes** |  | **No** | **Yes** |  |
| **Plaque fibrous volume (%)** | 40.0 (20.5) | 39.1 (17.9) | 0.504 | 40.1 (20.2) | 37.3 (17.5) | 0.054 |
| **Plaque fibrofatty volume (%)** | 4.4 (5.9) | 4.7 (5.9) | 0.069 | 4.4 (5.8) | 5.31 (6.8) | 0.053 |
| **Plaque necrotic core volume (%)** | 2.2 (4.8) | 2.5 (5.9) | 0.024 | 2.3 (5.1) | 2.5 (4.8) | 0.039 |
| **Plaque dense calcium volume (%)** | 53.3 (24.1) | 53.4 (22.8) | 0.988 | 53.1 (23.9) | 54.7 (23.3) | 0.330 |

**MACE – major adverse cardiovascular events, N = number of patients; values are shown as means and standard deviations (±SD)**
