# Supplementary figures and images for: From plaque to event: coronary plaque burden and morphology in predicting adverse cardiovascular outcomes
Source: Front Cardiovasc Med. 2026 Feb 2;13:1761012. doi: 10.3389/fcvm.2026.1761012 (PMC12907141; doi:10.3389/fcvm.2026.1761012)

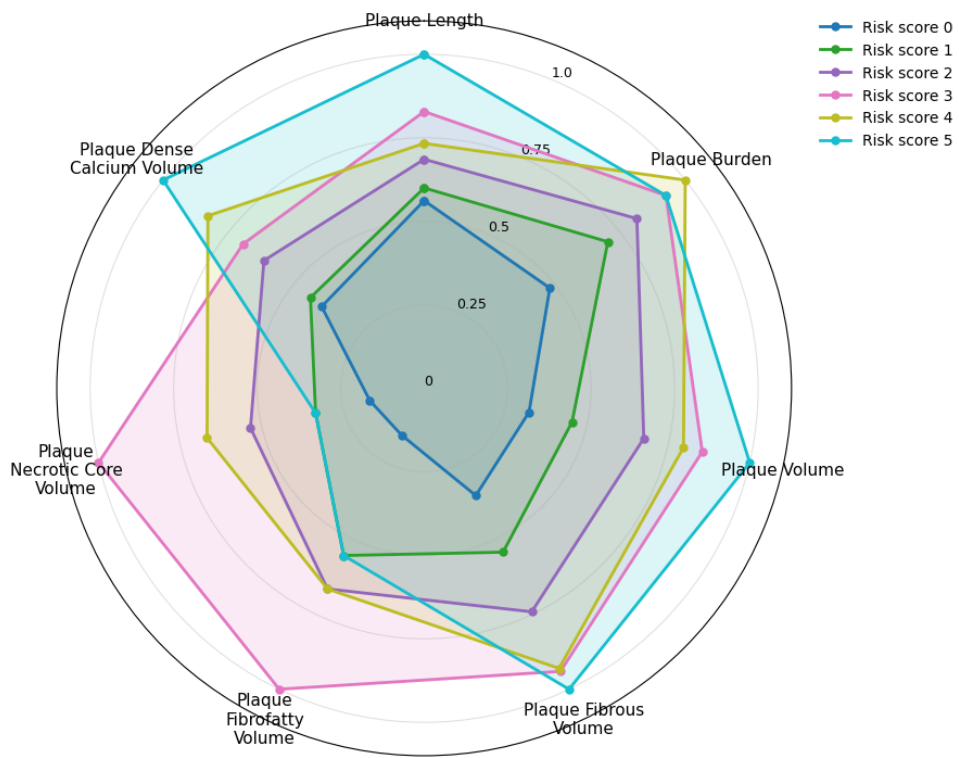

**Supplementary Figure 1. Radar chart of plaque parameters across different risk scores.**

Supplement: Supplementary file 2 [file Image1.pdf]
